# Buildings Contemplated

**Published:** 1914-02-21

**Authors:** 


### Buildings Contemplated.

Ashby-de-la-Zouch.—The Guardians are about to obtain plans and estimates for two children's homes, and have resolved to apply to the Local Government Board for sanction to purchase property in New Burton Road, at the cost of £1,350, for the erection of the same.

Basford.—The Local Government Board has authorised the Guardians to borrow £6,500 for the building of children's homes.

Bucknall.—The Local Government Board has agreed, subject to the approval of the Treasury, to grant £3,600 towards the cost of enlarging the fever hospital at Bucknall, belonging to Stoke-on-Trent and Stoke Rural Joint Hospital Board.

Bulwell.—The Basford Board of Guardians have decided to build homes for children at a cost of £6,500.

Bury St. Edmund's.—The new infirmary, which was built at a cost of £3,600, has been formally opened.

Cannock Chase.—The Staffordshire, Wolverhampton, and Dudley Joint Committee for Tuberculosis have resolved to erect a sanatorium, and a sub-committee has been appointed to view sites and obtain estimates, and to report thereon to the committee.

Canterbury.—Mr. W. J. Jennings, St. Margaret's Street, Canterbury, is architect for extensions to Canterbury Mental Hospital; an application by the Corporation for sanction to a loan of £25,000 to carry out the work has been the subject of a recent inquiry by the Local Government Board.

Chester-le-Street.—The Guardians have resolved to purchase a site for building children's cottage homes.

Chester-le-Street.—The Joint Hospital Board have under consideration plans for certain improvements at the hospital at an estimated cost of £2,500.

Coleraine.—The Londonderry County Council has resolved to provide a tuberculosis dispensary, at an estimated cost of £2,299.

Eastwood.—The Middlesex County Council has agreed to purchase about ninety acres of land at Eastwood, near Southend, at a cost of £3,562, for a county sanatorium.

Ecclesall.—The Ecclesall Guardians have decided to build a hospital for children at a cost of some £5,800, comprising sixty-four beds. Mr. J. Givie is chairman of the building committee.

Gilroes.—The Leicester Town Council has resolved to erect a sanatorium at Gilroes, adjoining the isolation hospital, at an estimated cost of £12,350.

High Carley.—The Lancashire County Council are inviting tenders for the erection of a sanatorium of about ninety beds at High Carley, near Ulverston. The architect is Mr. Dean J. Brundrit, County Square, Ulverston, from whom quantities and specifications may be obtained.

Meigh.—The Newry Guardians propose to build a dispensary, together with a residence for the medical officer, at an estimated cost of £1,000.

Newcastle-on-Tyne.—An additional wing to the workhouse, erected at a cost of £21,587, has been formally opened. The architect was Mr. E. Bowman, of County Chambers, Newcastle.

Roughton Heath.—The Erpingham Guardians are considering plans for the erection of an isolation hospital administrative block. The cost is estimated at £543.

Stoke-on-Trent.—The Guardians have resolved to apply to the Local Government Board for sanction to borrow £5,578 for a children's hospital.

Trowbridge.—Messrs. G. T. Hine and Pegg, of 35 Parliament Street, S.W., are architects for the proposed additions at the county mental hospital at Devizes. In regard thereto an inquiry has recently been held by the Local Government Board into an application of the Wiltshire County Council for sanction to a loan of £30,000 to cover the cost.

Wilts.—The Wiltshire County Council has sanctioned an extension of the county mental hospital, at an estimated cost of £30,000.

At the Cumberland Infirmary, Carlisle, there is a vacancy for a resident medical officer at a salary of £80 to £100.

